# In vivo study on borophene nanoflakes interaction with* Tenebrio molitor* beetle: viability of hemocytes and short-term immunity effect

**DOI:** 10.1038/s41598-023-38595-8

**Published:** 2023-07-21

**Authors:** Elżbieta Czarniewska, Krzysztof Sielicki, Klaudia Maślana, Ewa Mijowska

**Affiliations:** 1 Department of Animal Physiology and Developmental Biology, Institute of Experimental Biology, Faculty of Biology, Adam Mickiewicz University in Poznań, Uniwersytetu Poznańskiego Str. 6, 61-614 Poznan, Poland; 2 Faculty of Chemical Technology and Engineering, Nanomaterials Physicochemistry Department, West Pomeranian University of Technology, Szczecin, Piastow Ave. 42, 71-065 Szczecin, Poland

**Keywords:** Nanoparticles, Entomology, Cell death and immune response

## Abstract

The family of graphene-based materials welcomed a new member, borophene, in 2014. Research on synthesis routes and experimental study on physicochemical and biological (especially in vivo) properties still is strongly desired in order to evaluate its practical potential as a drug delivery-system. The effect of two-dimensional borophene nanoflakes on cells, systems and the entire animal organism has not been studied so far. Therefore, we investigated in vivo its biocompatibility with hemocytes in the *Tenebrio molitor* as a model organism. Short-term studies demonstrated that borophene nanoflakes at doses of 0.5, 1 or 2 µg of nanoflakes per insect did not induce hemocytotoxicity. Hemocytes exposed to nanoflakes showed morphology, adhesiveness and ability to form filopodia as in the control hemocytes. A detailed study indicates that borophene nanoflakes do not: (i) generate intracellular reactive oxygen species in hemocytes, (ii) affect the mitochondrial membrane potential and (iii) interfere with phagocytosis. Therefore, this contribution presents new in vivo insights into the group of two-dimensional materials which are one of the most promising materials for biomedical applications owing to their special structure and unique properties. However, long-term studies in insects and other animals are still necessary to confirm that borophene is biocompatible and biologically safe.

## Introduction

The development of nanomaterials has increased significantly in recent years, where different morphological structures can be distinguished: zero-dimensional (0D), one-dimensional (1D), two-dimensional (2D), and three-dimensional (3D). The most popular 2D structure, graphene, brought much attention to those two-dimensional architectures, which pushed the development and fabrication of other new materials—transition metal dichalcogenides (TMD), graphitic carbon nitride (gCN), hexagonal boron nitride (hBN), black phosphorous (BP) and so on. Graphene in the form of graphene oxide (GO) has broad potential applications in the fields of drug delivery, bioimaging, biosensing, or even tissue engineering^1^. However, it was proved that GO causes cytotoxicity, where it enters the cytoplasm and nucleus of cells leading to induced cell apoptosis. What is more, it also accumulates in the kidney and lung tissues and is difficult to remove^2–4^. Reduced GO due to the change of GO amphiphilic structure, led to difficulty in lipid partitioning, suppressing the hemolysis. It is clear that for graphene-based structures toxicity is highly dependent on their size, functional groups and lateral size^5^. The in vivo toxicity tests also prove the correlation between graphene structural properties and the concentration of the doses and entry points into living organisms. In contrast to graphene derivatives, the TMD show lower cytotoxicity when we exposed human lung epithelial cells (A549) to them. MoS_2_, WS_2_ and WSe_2_ presented low toxicity even at high concentrations (200 µg/mL)^6^. In vivo tests processed on mice showed that MoS_2_ is biocompatible and can be used in tumor treatment therapy^7^. MoS_2_ can be also utilized due to its compatibility as a biodegradable biosensor^8^. Furthermore, a size-dependent study on in vitro biocompatibility of graphitic carbon nitride proved that 10 nm and 160 nm are biocompatible. However, the gCN with a size of 20 nm showed the lowest cell viability. The gCN agglomerated mostly around the nuclei, nevertheless, it did not penetrate^9^. Another member of the 2D family—hexagonal boron nitride (hBN) (~ 120 nm in diameter) did not cause any lung damage at low doses. However, in other organs, when the dose was 1600 µg/kg, it caused damage to the lung, liver, kidney, heart or spleen^10^. BP can also be utilized as a successful alternative to harsh medicine in chemotherapy. It was presented that BP killed cancerous cells (HepG2) and was biocompatible with normal cells (QSG-7701). Therefore, BP could be utilized as an inorganic tool in less harmful cancer treatment^11^. It is clear that many factors (such as lateral size, surface properties, functional groups on the surface as well as different doses) impact the biocompatibility and toxicity of 2D nanomaterials. Nevertheless, it is crucial to test the 2D structures in both in vitro and in vivo experiments. Recently, discovered 2D members such as borophene should be cautiously investigated for their potential biocompatibility or toxicity. However, there is still room in the state of the art for research dedicated to borophene nanoflakes in their pristine form, especially regarding in vivo tests.

Borophene, which was first mentioned in 2014, is a new monoelemental 2D supermaterial that has attracted extensive attention from researchers due to its excellent chemical, electrical, mechanical and thermal properties ^12^. This new material has been first synthesized by two individual groups: Mannix et al*.*^13^ and Feng et al*.*^14^ by deposition at the silver substrate in UHV conditions from a high-purity solid substrate in 2015. It has been widely considered a promising nanomaterial for its potential application in the fields of electronic equipment and biomedicine^15–22^. It is a promising drug delivery and theranostic material due to its material properties and fluorescent and photoacoustic contrast for imaging, in addition to the photothermal and photodynamic therapeutic properties^21^. Borophene can be functionalized with fluorescent molecules for in vitro or in vivo fluorescence imaging to track the absorption pathways and cellular localization of borophene-based nanosystems and, consequently, to accurately locate neoplastic tumors^23,24^. For this reason, it is necessary to understand its interaction with the organism of animals and humans. Recently, the antibacterial activity of borophene nanoplatelets^25^ against *Staphylococcus aureus*, *Pseudomonas aureginosa*, *Escherichia coli* and antifungal activity against *Candida albicans* and *Aspergillus brasiliensis* has been also demonstrated. However, the mechanism of inhibitory activity against bacterial and fungal pathogenic microorganisms of borophene is not revealed so far^25^. Therefore, it is clear that the current state of the art does not provide fundamental knowledge on the biological activity of borophene in vitro and in vivo on cells, tissues, organs and the organism of animals. It has been only suggested that borophene may exert a cytotoxic effect on cells due to its highly reactive edges which may cause increased reactive oxygen species (ROS) generation^21,25^.

Therefore, in this study, we examined in vivo effect of borophene nanoflakes on the viability and function of the insect's immunocompetent cells (hemocytes) and their ability to produce ROS in these cells. The tests of the biocompatibility of borophene nanoflakes were conducted on the insect *Tenebrio molitor*^26^, which is a convenient experimental model for comprehensive in vivo studies of the effects of nanoparticles on various physiological parameters crucial for animal life^27–29^. Furthermore, the insect hemocytes present numerous structural and functional similarities to mammalian white blood cells which makes it even more interesting to study. Both insect hemocytes and mammalian leukocytes play a crucial role in defending the organism against pathogens. Neutrophils, macrophages, some dendritic cells of mammals and the insect plasmatocytes and granulocytes are all capable of phagocytosis. They can engulf and digest foreign particles, such as pathogens or cellular debris, as part of the cellular immune response^30^. Hemocytes in insects and certain mammalian leukocytes, such as neutrophils and macrophages, produce and release antimicrobial peptides (AMPs) to combat infections. Although the specific AMPs may differ, the general function of antimicrobial defence is conserved. Both insects and mammals possess Toll-like receptors expressed on their hemocytes/leukocytes which play a critical role in recognizing conserved molecular patterns present in pathogens, triggering immune responses^30^. Some leukocytes and hemocytes have also the ability to modulate immune responses, they release various pro-inflammatory molecules, such as cytokines and chemokines, which recruit and activate other immune cells to the site of infection or injury. They participate in tissue repair and wound healing processes contributing to clearing cellular debris, promoting tissue regeneration, and modulating the repair process through the secretion of growth factors and extracellular matrix components^31^_._

Here, short-term investigations revealed that borophene nanoflakes at different doses (0.5, 1 or 2 µg of nanoflakes per insect) did not induce hemocytotoxicity. Hemocytes exposed to the presence of borophene indicated morphology, adhesiveness and ability to form filopodia the same as in the reference hemocytes. What is also crucial borophene nanoflakes significantly increased the reducing power of hemocytes, did not generate intracellular reactive oxygen species (ROS) in hemocytes, did not affect the mitochondrial membrane potential and did not interfere with phagocytosis.

## Results and discussion

**Figure 1:**
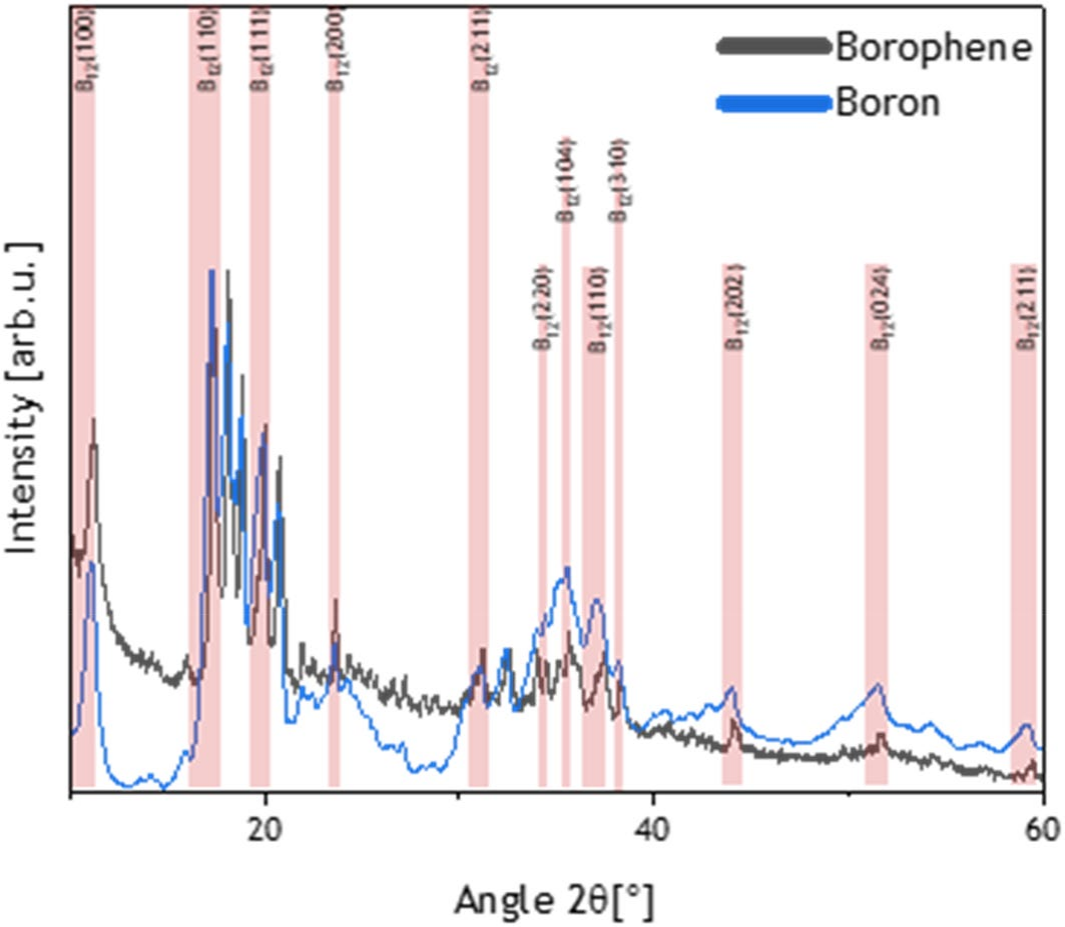
XRD diffractogram of bulk boron (blue) and borophene (dark grey).

Firstly, the bulk boron and synthesized borophene flakes were studied via X-Ray Diffraction (XRD) presented in Fig. 1. The structure of bulk boron is mostly composed of B_12_ icosahedron, leading to different structures based on basic unit variations^32^. XRD diffractograms indicate that both structures represent the B_12_ reflexes—which is described as the basic unit of few-layered borophene, ~ 2Θ: 11°, 17.3°, 20.7°, 23.5°, 31°, 34.5°, 35.5°, 37°, 38.2°, 44°, 51.5°, 59°. The sonochemical process of exfoliation provides some structural changes. Bulk boron is a rather amorphous structure with broad unsharp reflexes, composed of B_12_; when exfoliated, the reflexes become sharper and more distinguishable. It can be also assigned to the more clear and more crystalline structure of borophene, without an amorphous phase. The shift of borophene reflexes to higher angular values can be assigned to the reduction of lattice parameters.

**Figure 2:**
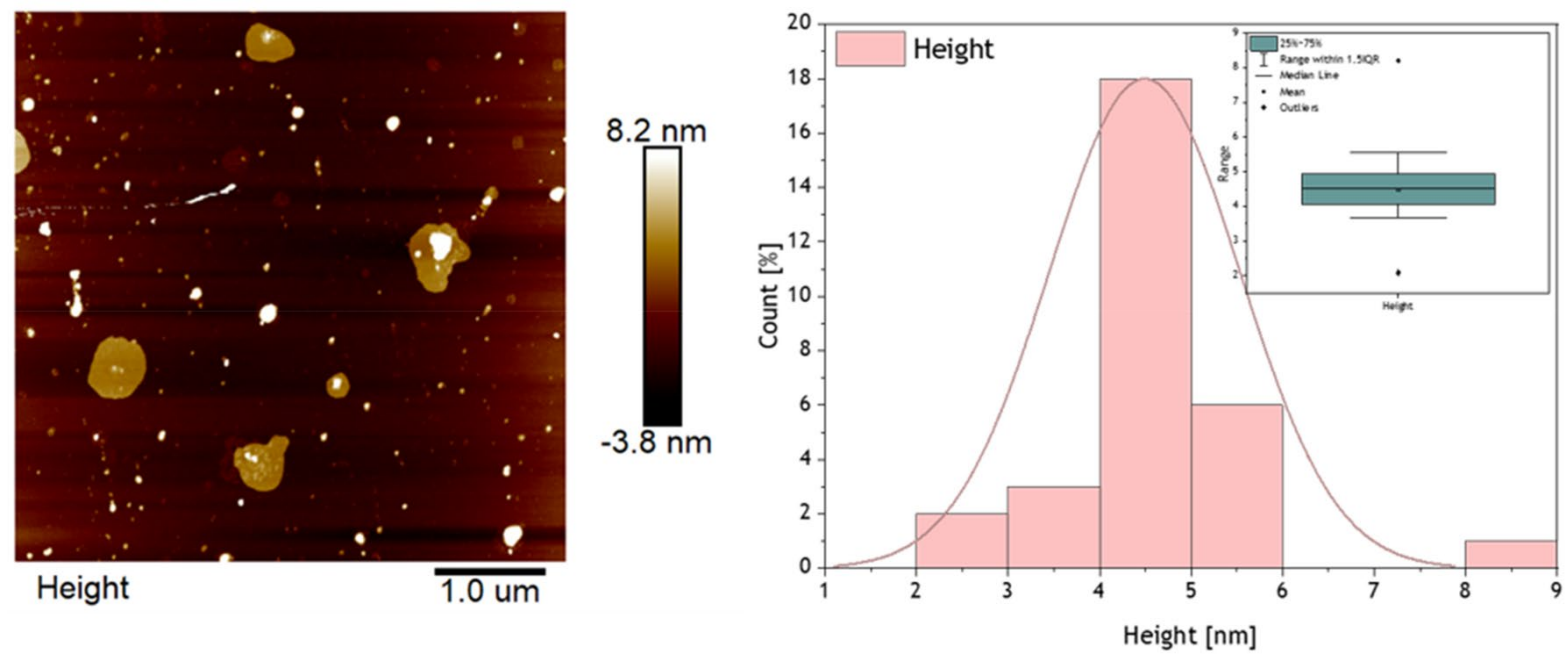
AFM image of borophene (left) and height distribution of borophene flakes (right) based on AFM profiles.

The lateral size and height of the borophene flakes were determined via an atomic force microscope (AFM) presented in Fig. 2.

The size of the flakes was in diameter from ~ 55 to ~ 549 nm. However, the height profiles of borophene flakes were distributed between ~ 2 and ~ 9 nm with the main phase of ~ 4.5 nm. Therefore, borophene flakes with an average height of ~ 4.5 nm corresponding to ~ 8 flakes will be studied in this research.

**Figure 3:**
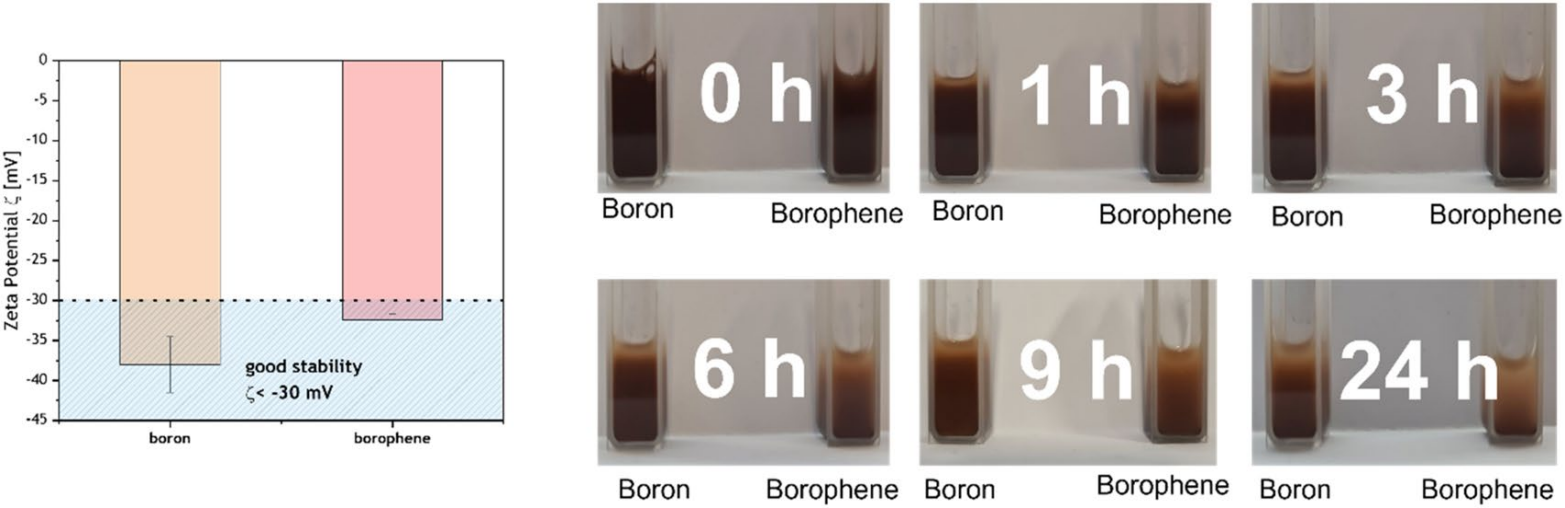
Zeta potential ζ (left) and dispersion stability measurement (right) of bulk boron and borophene.

The zeta potential (ζ) measurement was performed to determine the surface charge and overall suspension stability (Fig. 3, left).

**Figure 4:**
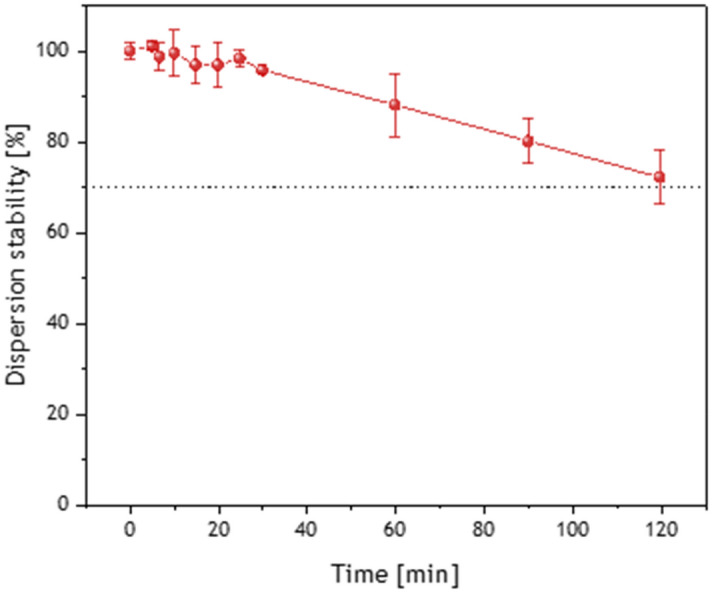
Stability of borophene dispersion in water (1 µg/µL) based on the absorbance of the solution (intensity of peak 554 nm).

For that, the homogeneous suspensions of bulk boron and borophene flakes in distilled water were prepared with a concentration of 1 mg/mL. All measurements were conducted with triple replication. The obtained value was −38 ± 3.5 mV and −32.4 ± 0.7 mV for bulk boron and borophene, respectively. Clearly, after the exfoliation the ζ value has decreased (statistically insignificant; p > 0.05) indicating that the surface of borophene changed compared to bulk boron. However, the values of both samples are typical for well-stabilized dispersion (for good stability ζ values > 30 mV or < -30 mV)^33^. The pictures of aqueous dispersions of borophene flakes and bulk boron (Fig. 3, right) prove good stability even after 24 h. Slightly better dispersion of bulk boron can be attributed to a higher ζ value, therefore, stronger repulsion forces. The stability of water-based dispersion of borophene was also estimated via an absorbance of the solution of nanomaterial with the concentration of 1 µg/µL in 2 h and it is presented in Fig. 4. It proves that borophene nanoflakes do not agglomerate significantly in this period what can be attributed to the affinity of borophene to water what is in agreement with zeta potential (ζ) measurement. The results indicate that pristine water-based suspensions of borophene flakes can be applied in our in vivo experiments without a need to add any stabilizers such as PEG which is widely used in graphene-based research on biological systems in order to avoid particles agglomeration.

The hemocytes of insects freely circulating in the open circulation system of insects show numerous structural and functional similarities to mammalian white blood cells^34^ and are very sensitive to the action of various biotic and abiotic factors^34^. For this reason, they are a perfect model for the in vivo detection of cytotoxic effects induced by nanomaterials^27–29^. There are a lot of reports in the current state of the art that graphene nanomaterials, after entering the body with blood and/or passing through physiological barriers, reach various organs, where they accumulate to a different extent. In these organs, they can cause acute and chronic inflammatory reactions through DNA damage, autophagy and necrosis, or they can induce cell apoptosis^35^. Therefore, it is crucial to verify the biological action of borophene as a new member of the 2D family and fill the gap in research on in vivo and in vitro effects of borophene nanoflakes on animals and human cells which is not studied so far.

**Figure 5:**
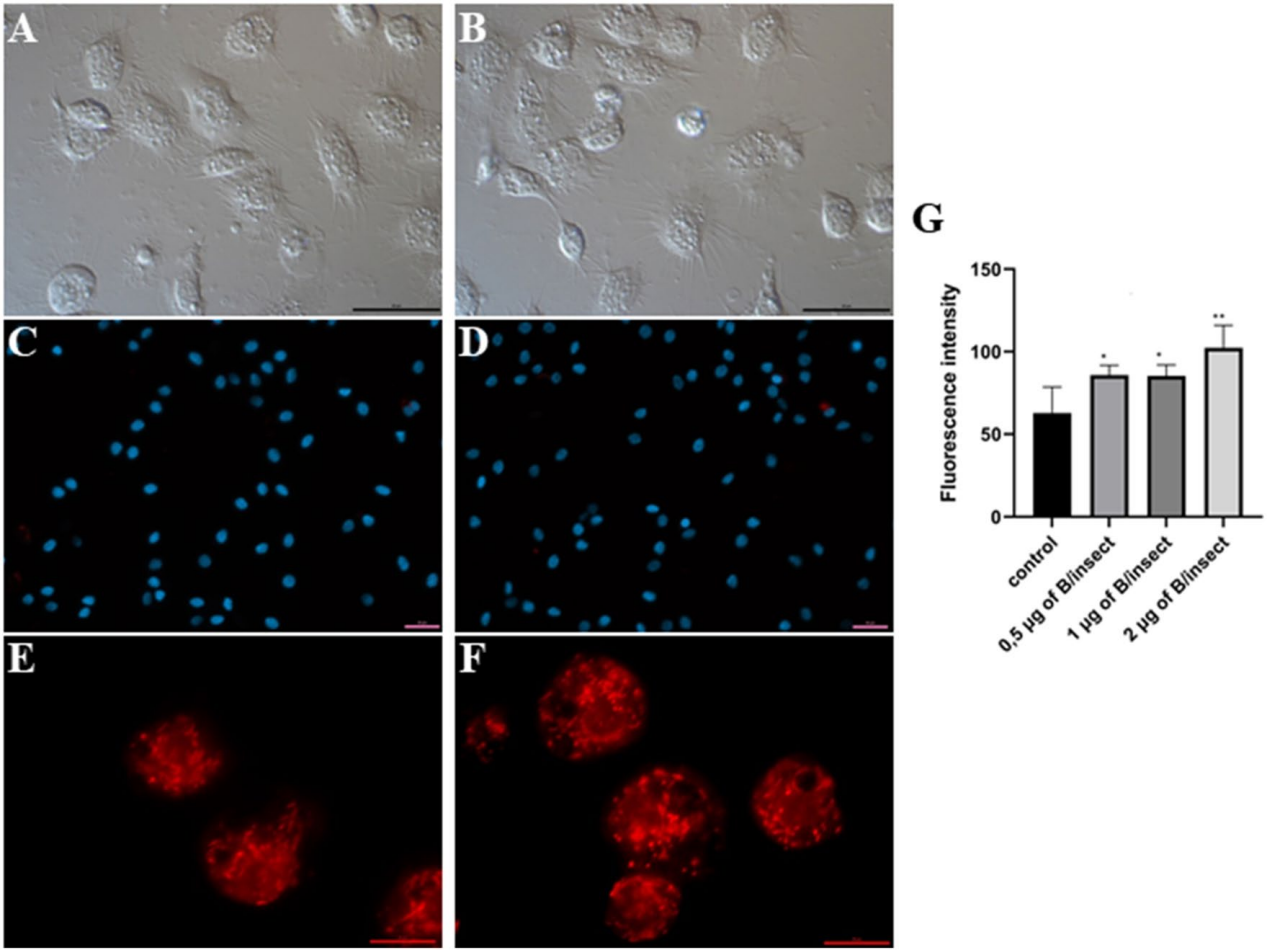
Morphology and viability of the *Tenebrio molitor* hemocytes two hours after saline or borophene nanoflakes (at a dose of 2 µg of nanoflakes per insect) injection. Nomarski's images showed no differences in the morphology and adhesion of hemocytes exposed to (**A**) saline and (**B**) borophene. Insect hemocytes injected with nanoflakes were just as capable of forming numerous and long filopodia as control cells. Fluorescence microscopy images of control and borophene-exposed hemocytes did not show induced apoptosis in hemocytes following saline (**C**) and borophene nanoflakes (**D**) injection; the active caspases (1–9) were stained with SR-VAD-FMK (no red = no active caspases) and DNA was stained with DAPI (blue). The MitoTracker Red CMXRos dye (red) was accumulated in the mitochondria of hemocytes exposed to saline (**E**) or borophene nanoflakes (**F**), showing that the borophene nanoflakes did not change the mitochondrial membrane potential and the mitochondria were active. Scale bars: 20 µm. (**G**) Alamar Blue cell metabolic assay for embedded hemocytes exposed to borophene at a dose of 0.5, 1 or 2 µg of nanoflakes per insect. Data are reported as mean ± SD (n = 6); asterisks denote differences between populations (*p < 0.02, **p < 0.05).

Firstly, to assess cell morphology and damage, hemocytes exposed to borophene were observed using phase-contrast microscopy (Fig. 5A, B) and fluorescence microscopy (Fig. 5C–F). The phase-contrast images of hemocytes exposed to borophene nanoflakes (at a dose of 2 µg nanoflakes per insect) demonstrated no changes in cell morphology. The nanoflake-exposed hemocytes had the same ability to adhere to coverslips and form long filopodia during adhesion as control hemocytes (Fig. 5A, B).

It is well-known that high concentrations of graphene disturb the dynamics and integrity of the plasma membrane during its internalization and consequently induce cell death. For example, it has been shown that exposure of MDA-MB-231^36^ and MCF-7 breast cancer cells, Panc-1 pancreatic cancer^37^ and GLC-82 lung cancer^38^ to high concentrations of GO induced a loss of membrane integrity of the cells as a result of invagination and disruption of the cell membrane at the site of interaction between graphene nanoflakes and the membrane^39^. Moreover, the internalized GO nanosheets were observed close to F-actin filaments of murine MC3T3-E1 preosteoblasts (A) and murine RAW-264.7 macrophages. The presence of GO nanosheets within the cytoskeleton caused cell cycle changes, apoptosis and oxidative stress in these cells^40^.

In our work, it is assumed that if the borophene nanoflakes disturb the integrity of the cell membrane of hemocytes and are located within the F-actin microfilaments, these changes should induce apoptosis in the borophene-exposed hemocytes. What is more, apoptosis involves, among others, DNA fragmentation and the activation of apoptotic marker enzymes, caspases, which recognize and cleave cellular target proteins leading to cell death. To give an answer to this hypothesis, a series of experiments have been conducted. Figure 5D clearly presents the fluorescence analysis of hemocyte viability using SR-VAD-FMK staining. It showed that the borophene nanoflakes did not induce the activation of caspases (1–9) and DAPI staining showed that there was no DNA fragmentation in the nuclei of hemocytes exposed to the nanoflakes. These results might suggest that the borophene nanoflakes do not accumulate within the cytoskeleton of hemocytes.

**Figure 6:**
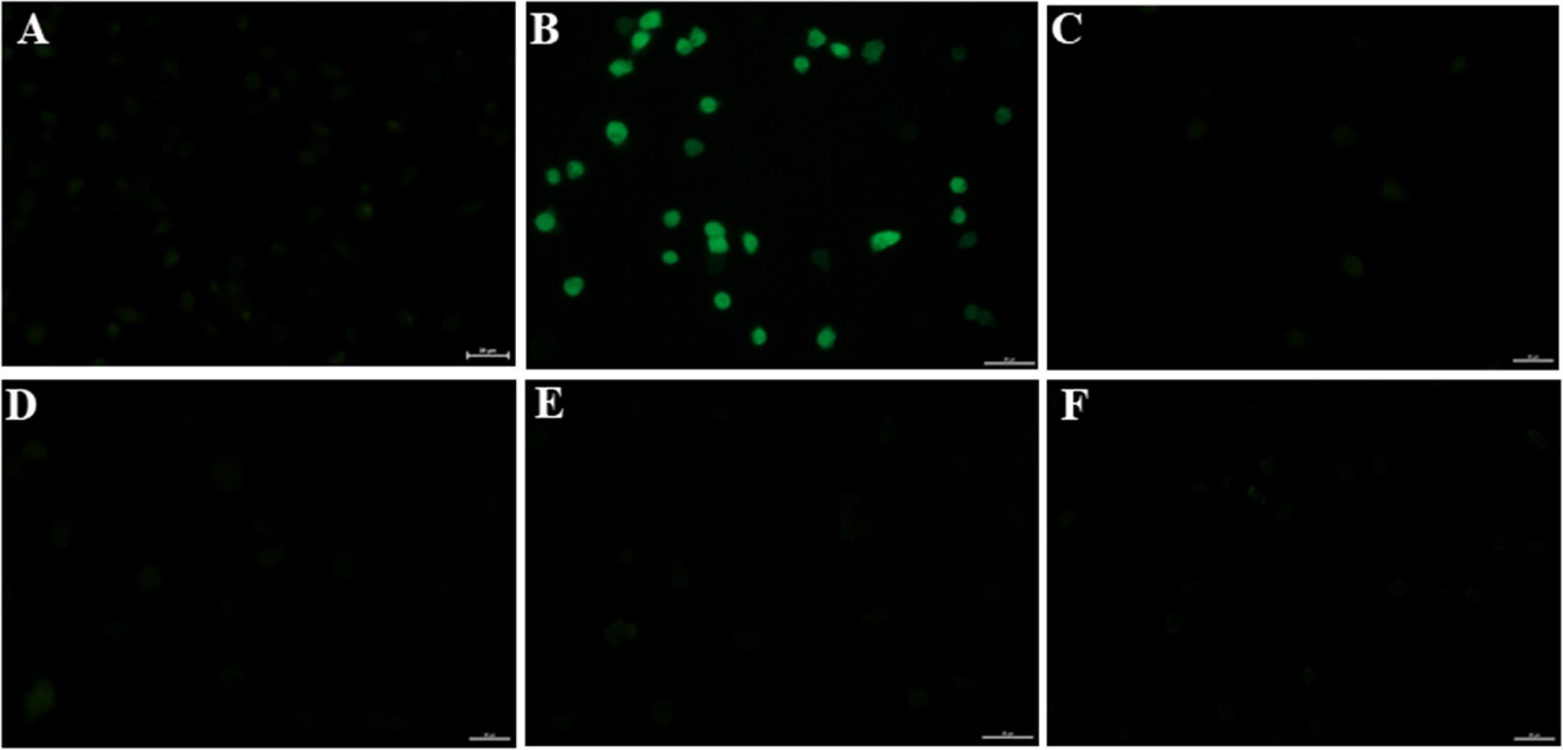
Intracellular ROS production in the *Tenebrio molitor* hemocytes 1 h after an injection of saline (**A**), injection of 40 nM hydrogen peroxide (**B**), injection of 0.25 (**C**), 0.5 (**D**), 1 (**E**) or 2 µg (**F**) of borophene nanoflakes per insect. The intensity of the green fluorescence of DCF indicates ROS concentration in the hydrogen peroxide-exposed hemocytes (positive control). DCFH_2_-DA staining of hemocytes exposed to saline (negative control) or borophene nanoflakes at the doses tested showed no ROS generation in these cells. Scale bars: 20 µm.

Due to the above-described observations, it is highly interesting to investigate the potential ROS production in the insect hemocytes exposed to the borophene nanoflakes. It has been shown that the main mechanism of cytotoxicity of two-dimensional graphene species is oxidative stress, which is the result of an increased level of ROS in the cell and damage of the cell membrane caused by nanoparticles^41–44^. In turn, the increased ROS production is one of the major factors leading to apoptotic cell death from ROS-induced lipid peroxidation, DNA damage and activation of caspases^44–48^. Mitochondria are recognized as the main source of ROS in cells^44,49^. To detect changes in mitochondrial membrane potential, a specific fluorescent dye accumulating exclusively in active mitochondria, MitoTracker Red CMXRos, was used. As shown in Fig. 5F, the borophene nanoflakes injected into the insect (at a dose of 2 µg of nanoflakes per insect) did not change the potential of the mitochondrial membrane, as evidenced by the accumulation of fluorochrome in mitochondria with normal morphology. This result indicates that the production of ROS in the mitochondria of hemocytes is not increased under exposure to the borophene nanoflakes injected into the insect at a tested dose. Subsequently, to confirm a lack of increase in intracellular ROS production in the borophene-exposed hemocytes a reductive reagent, DCFH_2_-DA, was applied. It reacts with ROS in cells and then is oxidized to fluorescent DCF. This study confirmed no increase in ROS levels in the hemocytes exposed to the borophene nanoflakes at the dose of 0.25, 0.5, 1 or 2 µg of nanoflakes per insect (Fig. 6C–F) when compared to control (Fig. 6A). On the other hand, ROS production was significantly increased in hemocytes exposed to hydrogen peroxide (Fig. 6B). Another indicator of cell viability, alamarBlue, is ideally configured to detect oxidation throughout the mitochondrial electron transport chain, and therefore, we used it to study the survival of hemocytes exposed to increasing doses of borophene nanoflakes (0.5,1 or 2 µg of nanoflakes per insect) and to confirm the lack of cytotoxicity of borophene nanoflakes in hemocytes (Fig. 5G). This study showed a dose-dependent increase in the natural reducing power of hemocytes exposed to increasing doses of borophene nanoflakes, demonstrating an increase in the viability of the borophene-exposed hemocytes compared to the control.

**Figure 7:**
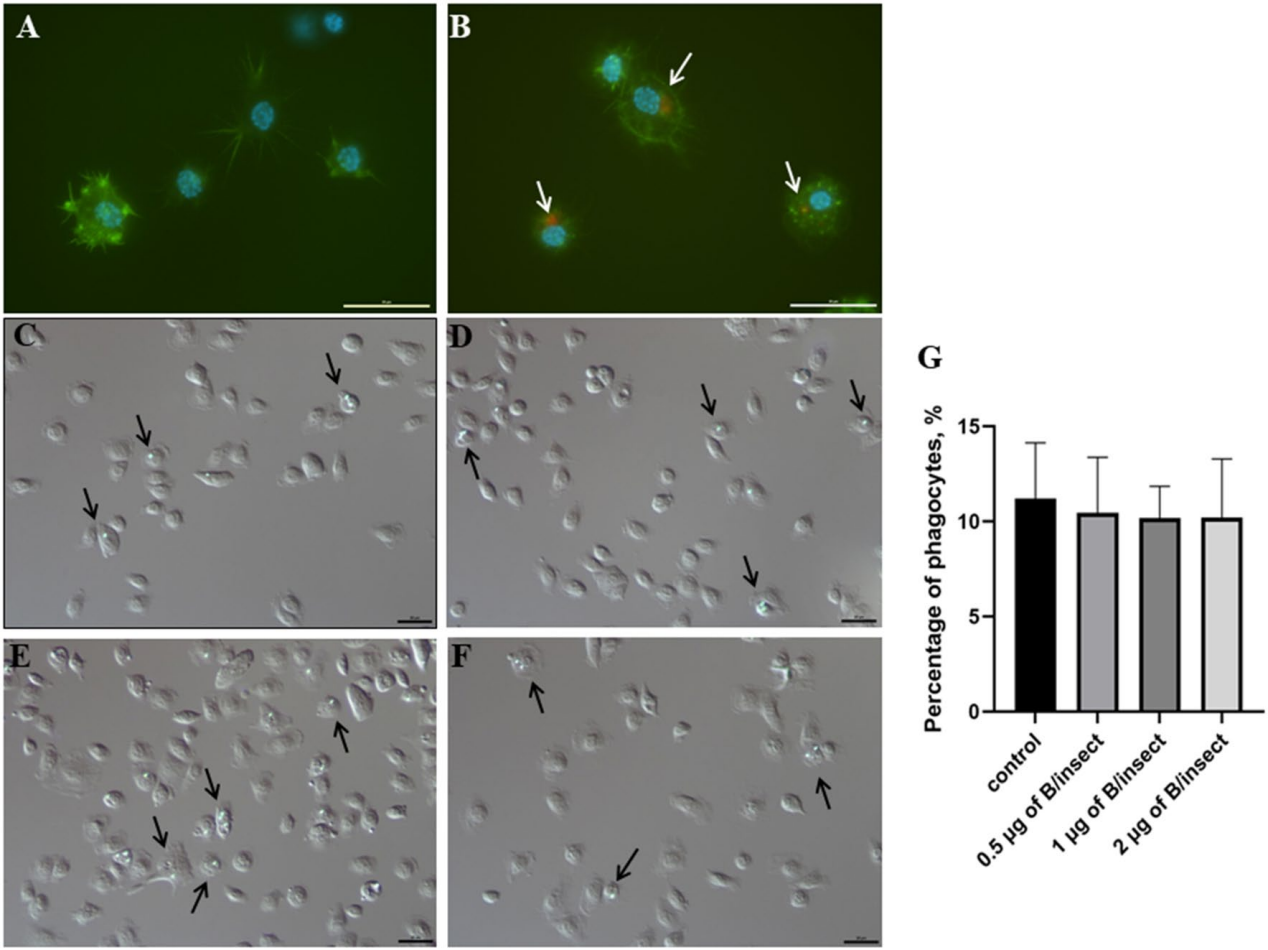
Phagocytic activity of the *Tenebrio molitor* hemocytes exposed to saline (**A**) and 2 µg of Alexa Fluor 647-borophene (**B**) or exposed to saline (**C**), 0.5 (**D**), 1 (**E**) and 2 µg (**F**) of borophene and then injected with fluorescent latex beads. Representative fluorescent microscopy images showing adhesive saline- or Alexa Fluor 647-borophene-exposed hemocytes stained with Oregon Green 488-phalloidin to visualize F-actin cytoskeleton and DAPI to visualize DNA (**A,B**), Alexa Fluor 647-borophene nanoflakes (red) engulfed by hemocytes indicated by white arrows (**B**). Representative Nomarski’s microscopy images show the phagocytosis of fluorescent latex beads by hemocytes of insects injected with saline and borophene nanoflakes (**D–F**). Black arrows show phagocytes with fluorescent latex beads (green). Scale bars: 20 µm. (**G**) The percentage of saline- and borophene-exposed hemocytes containing latex beads was measured (mean ± SEM, Student's t-test).

It has been demonstrated that the internalization of the graphene-related nanomaterials into cells is strongly influenced by particle size; the protein-coated GO nanosheets of large and small sizes were taken up by cells primarily by phagocytosis and clathrin-mediated endocytosis, respectively^50^. The cellular processes that mediate phagocytosis are driven by a diverse set of molecular mechanisms and biophysical implications of cytoskeleton assembly and remodelling in the phagocyte membrane^51^. Here, the lateral size of the borophene nanoflakes used in the experiments ranged from ~ 55 to ~ 550 nm and its thickness was ~ 4.5 nm (Fig. 4) and when the Alexa Fluor 647-borophene nanoflakes solution was injected into the insect's hemocoel, the fluorescent nanoflakes were effectively phagocytosed and aggregated in the cytoplasm of hemocytes (Fig. 7B) in contrast to hemocytes of control insects injected only with saline (Fig. 7A). Subsequently, in the experiment showing the influence of the borophene nanoflakes on phagocytosis of another abiotic target, it is demonstrated that these nanoflakes (at all tested doses) did not impair the phagocytosis of latex beads (Fig. 7C–G).

These results suggest that during phagocytosis of the borophene nanoflakes or phagocytosis of the latex beads by hemocytes previously exposed to the borophene nanoflakes, the nanoflakes do not interfere with cytoskeleton remodelling and the formation of filopodia, which are necessary precursors of phagosome formation^51^. The lack of changes in the viability of the nanoflake-exposed hemocytes, their ability to form long filopodia during the adhesion and to phagocytose of the latex beads confirm our hypothesis about the non-accumulation of the borophene nanoflakes in the F-actin network forming the cytoskeleton of hemocyte. Interestingly, this is contrary to graphene-based research. It is reported that microsized GO induced much stronger inflammation responses, while nanosized graphene sheets showed better biocompatibility in macrophages^52^. Another study demonstrated that graphene after internalization was accumulated in the cytoplasm, perinuclear space and cell nucleus of mouse macrophages inducing cytotoxicity by (1) increasing intracellular ROS (2) reducing mitochondrial membrane potential and (3) inducing apoptosis by activating the mitochondrial pathway^53^. In turn, our previous studies in *T. molitor* showed that injection or topical application of nanodiamonds or the hydroxyl-functionalized exfoliated hexagonal boron nitride nanoflakes (h-BN-OH-n) also did not affect adhesion, viability and the complex function of cell membrane of hemocytes and did not interfere with the phagocytosis of latex beads. However, the long-term immunoassay demonstrated that h-BN-OH-n impaired the nodule formation in the hemocoel of insects after bacterial challenge as a result of the h-BN-OH-mediated decrease in the ability of hemocytes to recognize bacteria, migrate to them or form macroaggregates around them^27,28^. This result suggests that the long-term study of the immune response of the borophene-exposed hemocytes is necessary to have a full answer on in vivo biocompatibility of this nanomaterial.

## Conclusion

To summarize, we fill the gap in research on in vivo interaction of 2D borophene nanoflakes with hemocytes of the *T. molitor* beetle revealing that the nanomaterial does not induce hemocytotoxicity in short-term studies at doses of 0.5, 1 or 2 µg of nanoflakes per insect. The cells' morphology, adhesiveness, ability to form long filopodia and viability were detected to be the same as in the control hemocytes. Moreover, the results indicate that borophene nanoflakes increased the reducing power of hemocytes and did not generate intracellular reactive oxygen species in hemocytes or affect the mitochondrial membrane potential. The test of the immunological activity of hemocytes demonstrated that the nanoflakes did not influence phagocytosis. Therefore, this contribution presents new insights into the group of 2D materials which are one of the most promising materials for biomedical applications owing to their special structure and unique properties. However, long-term in vivo studies in insects and other animal models are still necessary to unequivocally confirm that 2D borophene is biocompatible and a biologically safe new nanomaterial for use in industry and medicine.

## Materials and methods

### Materials

Boron powder (B, CAS: 7440-42-8) was purchased from Sigma Aldrich (USA).

### Characterization

The zeta-potential was measured on a Zeta Sizer (ZS Nano ZEN 3600, Malvern) to determine the surface potential. Atomic Force Microscopy [AFM MultiMode 8 (Bruker)] provide information about the thickness and lattice size of exfoliated materials. The analysis of phase composition and crystallinity of materials was executed via Aeris diffractometer (Malvern Panalytical) and Cu-Kα radiation (λ = 1.544 Å). The UV–vis was performed using a Jasco (Japan) spectrometer.

### 2D borophene nanosheets preparation and fluorescent dye labelling

The borophene was synthesized from bulk boron via sonochemical method presented elsewhere^54^. Briefly, 300 mg of boron was dispersed in 80 mL of acetone and kept under ultrasonic sonotrode (Sonics&Materials, 20 kHz) for 24 h. After that, the solution was centrifuged at a speed of 5000 rpm for 3 min. The supernatant was collected and dried firstly at 60 °C for 6 h and at 100 °C for 12 h in a vacuum dryer.

To show that borophene nanoflakes can be engulfed by hemocytes the nanomaterial was labelled with a fluorescent dye. A solution of 1 µg/mL Alexa Fluor 647 (Thermo Fisher Scientific) in dimethylformamide (DMF, Sigma Aldrich) was prepared. 10 mg of borophene was mixed with 10 mL of Alexa Fluor 647. After 24 h, the material was centrifuged (5 min. at 5000 rpm), washed in DMF and water, and dried overnight at 35 °C.

### Preparation of the borophene nanoflakes solution

The borophene or the Alexa Fluor 647-borophene was dissolved in physiological saline for *Tenebrio* to yield stock solutions of 1 mg/mL. The prepared stock solutions were stored at − 30 °C, and the working solutions were prepared in physiological saline just before use. The borophene solutions with different concentrations were sonicated for 15 min at 80 W before use to avoid possible aggregates.

### Insects

A stock culture of *T. molitor* was maintained at the Department of Animal Physiology and Developmental Biology, Institute of Experimental Biology, Adam Mickiewicz University in Poznań, Poland. All beetles used in our experiments were derived from parents that were less than 1 month old. The control and experimental groups were maintained in separate plastic boxes in a climate chamber at a constant temperature of 26 °C, with a relative humidity of 60 ± 5% and a photoperiod of 12 h of light and 12 h of darkness. The experiments were carried out on fifteen 4-day-old adult insects for each treatment. The insects were injected with a borophene nanoflake solution at a dose of 2 µg of nanoflakes per insect (hemocyte adhesion, hemocyte apoptosis, active mitochondria assay, phagocytosis of Alexa Fluor 647-borophene nanoflakes), 0.5, 1 or 2 µg nanoflakes per insect (phagocytosis of the latex beads and alamarBlue assay) and 0.25, 0.5, 1 or 2 µg nanoflakes (ROS assay).

### In vivo biocompatibility tests

#### Hemocyte adhesion

The 4-day-old beetles were anaesthetized with CO_2_, washed in distilled water and disinfected with 70% ethanol. Borophene nanoflakes solution was injected (2 µL, in a dose of 2 µg per insect) through the ventral membrane between the second and the third abdominal segments towards the head, with a Hamilton syringe (Hamilton Co., Bonaduz, Switzerland). The control insects were injected with the same volume of physiological saline. All injections were performed in sterile conditions. Before hemolymph collection, the beetles were anaesthetized again with CO_2_, washed in distilled water and disinfected with 70% ethanol. Both control and borophene-injected insects were taken three hours after injection, and the hemocytes were prepared. Briefly, hemolymph samples (5 µL) were collected with ‘end to end’ microcapillaries (Drummond Scientific, Broomall, PA, USA), after cutting off a tarsus from a foreleg. Hemolymph was diluted in 20 µL of ice-cold physiological saline containing anticoagulant buffer (4.5 mmol L^−1^ citric acid and 9 mmol L^−1^ sodium citrate) in a 5:1 v/v ratio. The hemolymph from the control and the nanoflakes-injected insects was dropped onto alcohol-cleaned coverslips coated with 7 µL 0.01% poly-l-lysine (Sigma P4707, St Louis, MO, USA). The hemocytes were allowed to settle (15 min, at room temperature) and the remaining fluid was then removed. The settled cells were washed twice with physiological saline and incubated in saline for 15 min. Fixation was achieved in 4% paraformaldehyde. The hemocytes were examined with a Nikon Eclipse TE 2000-U fluorescence microscope equipped with Nomarski optics to study the hemocyte adhesion, the images were documented with a Nikon DS-1QM digital camera.

#### The hemocyte apoptosis

The cytotoxicity of borophene has been evaluated on insect hemocytes using a hemocyte bioassay in vivo as previously described^55^. Briefly, the adult beetles were split into four experimental groups—including a saline-treated control group and three borophene-treated groups. The experimental groups of beetles were injected with the borophene solution (2 µL) at a dose of 0.5, 1 or 2 µg of nanoflakes per insect, respectively, using a Hamilton syringe (Hamilton Co., Bonaduz, Switzerland), whereas the control group of beetles was injected with the same volume of saline. One hour after injection hemolymph samples (5 µL) were collected; the hemocytes were prepared and stained for the detection of active caspase using an inhibitor of caspase (1–9) activity (a sulforhodamine derivative of the valyl-alanyl-aspartic acid fluoromethyl ketone, SR-VAD-FMK; AK-115, BIOMOL, Plymouth Meeting, PA, USA) and visualization of hemocyte nuclei using DAPI. The hemocytes were examined with a Nikon Eclipse TE 2000-U fluorescence microscope to detect apoptosis and the images were documented with a Nikon DS-1QM digital camera.

### Assay for active mitochondria

MitoTracker Red CMXRos (Molecular Probes, Eugene, OR, USA) is a red fluorescent dye that stains mitochondria in live cells. The anaesthetized and disinfected 4-day-old experimental beetles were injected with the borophene solution (2 µL) at a dose of 2 µg of nanoflakes per insect, whereas the control group of beetles was injected with the same volume of saline. Then, 20 min after injection both groups of beetles were injected with 1 µM MitoTracker Red CMX Ros solution (2 µL) and 40 min after incubation the hemolymph samples were collected and the hemocytes were placed onto alcohol-cleaned coverslips coated with 7 µL 0.01% poly-l-lysine for 15 min, washed with saline, incubated with 100 nM MitoTracker Red CMX Ros solution for 40 min, washed with saline and fixed in 4% paraformaldehyde. The hemocytes were examined with a Nikon Eclipse TE 2000-U fluorescence microscope to detect mitochondria and the images were documented with a Nikon DS-1QM digital camera.

### alamarBlue hemocyte viability assay

Hemolymph (3 μl) of insects injected with 2 μl nanoflakes solution at a dose of 0, 5, 1 or 2 µg of borophene per insect was collected 1 h after injection and it was diluted in 27 µl of anticoagulant solution. Then, 5 µl of diluted hemolymph were mixed with 5 µl of trypan blue solution 0.4% (Sigma-Aldrich) to measure the numer of hemocytes in 1 µl of hemolymph using LUNA-II (Logos Biosystems Inc.). Hemocyte viability was measured using the alamarBlue assay (Invitrogen) using a resazurin-based solution that functions as a cell health indicator by using the reducing power of living cells to quantitatively measure viability. Hemolymph probes (10 µl) were plated in a 96-well (black flat bottom) Nunclon delta microplate (Thermo Fisher) and alamarBlue solution were added and incubated for 4 h (37 °C, 5% CO_2_). Upon entering living cells, resazurin is reduced to resorufin, a compound that is red in color and highly fluorescent. Fluorescence intensity was read using a aparat microplate spectrofluorometer—Tecan Infinite 200 PRO (Tecan AG) at excitation 530 nm and emission 590 nm. The fluorescence intensity of each hemolymph probe was calculated for 1000 hemocytes.

### Assay for evaluation of ROS

Intracellular ROS levels were assayed using 2′,7′-dichlorodihydrofluorescein diacetate (DCFH_2_-DA), which is cleaved by nonspecific esterases to generate 2′,7′-dichlorodihydrofluorescein (DCFH_2_), and quantitatively oxidized by ROS to generate fluorescent 2′,7′-dichlorofluorescein (DCF)^56^. In the present study, the 4-day-old beetles were injected with the borophene nanoflakes solution (2 µL) at a dose of 0.25, 0.5, 1 or 2 µg of nanoflakes per insect, whereas the control group of beetles was injected with the same volume of saline or 40 nM solution of H_2_O_2_. One hour after the injection of saline, H_2_O_2_ solution or nanoflakes, the hemolymph samples (2 µL) were collected and the hemocytes were prepared and used for ROS detection. For this purpose, the hemocytes were stained with 10 µM of 2′, 7′-dichlorofluorescein-diacetate (DCFH-DA) for 20 min, at room temperature in the darkness. Live-cell imaging was performed using a Nikon Eclipse TE 2000- U fluorescence microscope. The images were taken with a Nikon DS-1QM digital camera.

### Phagocytosis assay

An in vivo phagocytosis bioassay was used to show the ability of hemocytes to engulf the borophene nanoflakes. In the first experiment, fluorescent Alexa Fluor 647-labelled borophene was used to investigate whether these nanoflakes would be phagocytosed by hemocytes after their injection into the hemolymph of the insect. Four-day-old *T. molitor* beetles were split into the control group and the experimental group and were anaesthetized, disinfected with 70% ethanol and washed in distilled water before saline or Alexa Fluor 647-borophene treatment. The control group of beetles was injected with 2 µL of physiological saline, whereas the experimental group was injected with 2 µL of Alexa Fluor 647-borophene solution, administering a dose of 2 µg of Alexa Fluor 647-borophene nanoflakes per insect. Hemolymph was collected from the insects one hour after injection. The injection, hemolymph collection and hemocyte preparation procedures were carried out according to previously described methods^29^. To visualize the F-actin, the hemocytes were stained with Oregon Green 488-phalloidin (ThermoFisher Scientific) and to visualize the cell nuclei, the cells were stained with DAPI (Sigma-Aldrich) solution. The preparations of hemocytes were examined under a Nikon Eclipse TE 2000-U fluorescence microscope to detect the presence of Alexa Fluor 647-borophene nanoflakes in the interior of hemocytes. Each experimental group consisted of fifteen insects.

Simultaneously with the same biotest, we studied whether, after phagocytizing the borophene nanoflakes, the hemocytes retained the ability to phagocytose another abiotic target—the fluorescent latex beads^27^. Briefly, adult beetles were split into four experimental groups—including the saline-treated control group and three borophene-treated groups. All insects were anaesthetized, and the control group was injected with physiological saline (2 µL), the first experimental group was injected with the borophene nanoflake solution (2 µL) in a dose of 0,5 µg nanoflakes per insect, the second group was injected with borophene nanoflake solution (2 µL) in a dose of 1 µg nanoflakes per insect and the third group received an injection 2 µg of borophene nanoflakes per insect. One hour after saline and nanoflakes exposure, the insects were anaesthetized again, disinfected and injected with 2 µL of the fluorescent latex bead suspension (diluted at a ratio of 1:1000 v/v in sterile saline; Sigma-Aldrich L1030). Hemolymph samples (5 µL) were collected one hour after the latex bead injection, and the hemocytes were prepared. The hemocytes were washed with saline and fixed with 4% paraformaldehyde for 10 min. Then, the hemocytes were washed again, mounted and examined with a Nikon Eclipse TE 2000-U fluorescence microscope equipped with Nomarski optics. Five fields of view were randomly chosen per glass slide and the percentage of hemocytes which engulfed fluorescent latex beads were calculated. Each experimental group consisted of fifteen insects.

### Statistical analysis

All data were presented as mean ± standard deviation. Statistical analyses were performed by using a Student’s *t*-test (Graphpad Prism 7.0, San Diego, CA, USA). *P* values less than 0.05 were considered statistically significant differences among the compared groups.

## Data Availability

The datasets used during the current study are available from the corresponding author upon reasonable request.
